# Acute Toxicity and Ecological Risk Assessment of Benzophenone and *N,N*-Diethyl-3 Methylbenzamide in Personal Care Products

**DOI:** 10.3390/ijerph13090925

**Published:** 2016-09-19

**Authors:** Hong-Qin Sun, Yang Du, Zi-Yang Zhang, Wen-Jing Jiang, Yan-Min Guo, Xi-Wu Lu, Yi-Min Zhang, Li-Wei Sun

**Affiliations:** 1 School of Energy and Environment, Southeast University, 2 Sipailou, Nanjing 210096, China; sunhongqin123456@163.com (H.-Q.S.); ngd2011dy@126.com (Y.D.); 2010jwj@sina.com (W.-J.J.); 220140548@seu.edu.cn (Y.-M.G.); xiwulu@seu.edu.cn (X.-W.L.); 2 Taihu Lake Water Environment Engineering Research Center (Wuxi), Southeast University, 99 Linghu Street, Wuxi 214000, China; 3 Department of Building, Civil, and Environmental Engineering, Concordia University, 1455 de Maisonneuve, Boulevard West, Montreal, QC H3G 1M8, Canada; zhangziyang1028@hotmail.com; 4 Research Center of Watershed Ecological Conservation and Water Pollution Control, Nanjing Institute of Environmental Sciences, Ministry of Environmental Protection of the People’s Republic of China, 8 Jiangwangmiao Street, Nanjing 210042, China

**Keywords:** benzophenone, *N*,*N*-diethyl-3-methylbenzamide, personal care products, acute toxicity, ecological risk assessment

## Abstract

Benzophenone (BP) and *N,N*-diethyl-3-methylbenzamide (DEET) are two chemicals often used in personal care products (PCPs). There is a lack of systematic ecotoxicological evaluations about the two chemicals to aquatic organisms. In the present study, the acute toxic effects on *Chlorella vulgaris*, *Daphnia Magana*, and *Brachydanio rerio* were tested and the ecotoxicological risks were evaluated. For BP, the 96-h half-maximal effective concentration (EC_50_) on *C. vulgaris* was 6.86 mg/L; the 24-h median lethal concentration (LC_50_) on *D. magana* was 7.63 mg/L; the 96-h LC_50_ on *B. rerio* was 14.73 mg/L. For DEET, those were 270.72 mg/L, 40.74 mg/L, and 109.67 mg/L, respectively. The mixture toxicity of BP and DEET, on *C. vulgaris*, *D. magana*, and *B. rerio* all showed an additive effect. The induced predicted no-effect concentrations (PNECs) for BP and DEET by assessment factor (AF) method are 0.003 mg/L and 0.407 mg/L, respectively. Both are lower than the concentrations detected from environment at present, verifying that BP and DEET are low-risk chemicals to the environment.

## 1. Introduction

Personal care products (PCPs) are one of the groups in emerging pollutants and are integral to people’s activities of daily life. Pedrouzo et al. [1] sub-classified PCPs into six categories: organic ultraviolet (UV) filters, antimicrobials, preservatives, musk fragrances, insect repellents, and siloxanes. These compounds are not usually considered in routine environmental monitoring, but are becoming of environmental concern because of their increasingly common use and the potential risk to human and ecosystem health. With the improvement in detection techniques, an increasing number of PCPs are being detected from different aquatic environments [2]. A study [3] also indicated that 2-Hydroxy-4-methoxybenzophenone (BP-3), one ingredient of PCPs, has been detected from the human body. Furthermore, the concentrations of BP-3 in females are higher than those in males, because females tend to use cosmetics more than males do. The result indicated that the risk of exposure to PCPs is related to human lifestyles. Predictably, with changes in lifestyles, the types and amounts of PCPs being used will gradually increase; therefore, the evaluations about the ecological risk and hazardous impact on human health of PCPs should be emphasized.

Benzophenone (BP) is a main member of UV filters, and is extensively used as a sunscreen agent [4] to protect skin and hair [5] from being damaged by UV rays. BP is also used in perfumes, as it can provide a sweet smell [6]. BP has been detected in various aquatic environments, including surface water, sediment [7], and urban sewage. BP can generally enter the body through breathing, diet, and skin contact. Toxicological studies [8] have shown that BP is regarded as the third-generation environmental pollutant (endocrine disruptors) because of its genetic toxicity and biological toxicity effects. However, research [5,9] on ecological toxic effects of BP remains scarce.

Diethyltoluamide (*N*,*N*-diethyl-3-methylbenzamide or DEET) is the main ingredient of insect repellents. DEET has been used as an insect repellent for a long time, and its consumption is very high. DEET has been detected in different aquatic environments [10], but there few ecological toxicity studies [11] on DEET have been reported.

To evaluate the ecological toxicity of BP and DEET, *Chlorella vulgaris*, *Daphnia magna*, and *Brachydanio rerio* were employed to conduct independent acute toxicity and further mixed toxicity tests. The three organisms used in the toxic test are representative ones for the 3 typical tropic levels in the aquatic ecosystem: *C. vulgaris* represented the autotrophs (primary producer); *D. magna* represented the primary consumer (predator); *B. rerio* represented the second consumer (second predator). 

All the experiments were conducted in accordance with Chinese National Standards: GB/T 21805-2008 (algae) [12], GB/T 16125-2012 (daphnia magna straus) [13], GB/T 27861-2011 (fish) [14]; and also methods in Analytical Methods for Water and Wastewater [15]. These methods basically referred to the OECD or ISO standards, except *C. vulgaris* is added as an algae candidate by the Chinese National Standards.

Based on the results of acute toxicity to the three organisms, the mixed toxicity of the two chemicals were tested, and then the ecological risk of BP and DEET were assessed by the predicted no-effect concentration (PNEC)—the maximum concentration at which the ecosystem is protected. The results will provide the scientific basis and reference value for formulating environmental criteria.

## 2. Materials and Methods

### 2.1. Preparation of Chemicals

Analytical purity grade BP was purchased from Sinopharm Chemical Reagent Co. Ltd. (Shanghai, China). DEET at 99% purity was purchased from Aladdin Industrial Corporation (Shanghai, China). Analytical purity grade dimethyl sulfoxide was purchased from Sinopharm Chemical Reagent Co. Ltd. and used as a cosolvent.

### 2.2. Experimental Biota for Toxicity Test

*C. vulgaris* (FACHB-8) was obtained from the Freshwater Algae Culture Collection, at the Institute of Hydrobiology, Chinese Academy of Sciences (Wuhan, China). The algae were cultured for three generations in the laboratory before the toxicity experiments. The algae cells in the logarithmic growth phase were used for toxicity experiments. The experiments were conducted in an illumination incubator to maintain the same condition: 2000–3000 lx, 25 ± 2 °C, pH 7.1, 12 h:12 h light–dark cycle.

*D. magna* were obtained from the Freshwater Algae Culture Collection at the Institute of Hydrobiology, Chinese Academy of Sciences (Wuhan, China). The *D. magna* were cultured in the laboratory and were tested by potassium dichromate before the toxicity experiments. The *D. magna* for the test were 6–24-h old. The experiments were conducted using an illumination incubator to maintain the same condition: 25 ± 2 °C, pH 7.0–8.0, 12 h:12 h light–dark cycle.

*B. rerio* (AB strains) were ordered from Nanjing YSY Biotech Company Ltd. (Nanjing, China). The average body length was 2.60 ± 0.20 cm, average body weight was 0.33 ± 0.06 g, and potassium dichromate was used to test the sensitivity of *B. rerio* before the toxicity experiments. The experiments were carried out at: 25 ± 2 °C, pH 7.0–8.0, 12 h:12 h light–dark cycle.

### 2.3. Experimental Design

#### 2.3.1. Ninety-Six Hours Acute Toxicity Experiments with *C. vulgaris*

During the acute toxicity experiments on *C. vulgaris*, BG 11 liquid medium and BP or DEET stock solution were used to prepare a serial concentration of solution (100 mL) in a glass flask (250 mL). The initial density of *C. vulgaris* was 10^6^ cells/mL, and the experiment lasted 96 h. Each concentration test, including cosolvent and blank control groups, was designed with three parallel samples. Before the experiment, microscopic counting and spectrophotometry were employed to obtain the linear correlation between the *C. vulgaris* cells density and absorbance at 680-nm wavelength. During the period of the experiment, the absorbance of *C. vulgaris* was measured every 24 h, then it was converted to cell density to calculate growth rates.

#### 2.3.2. Twenty-Four Hours Acute Toxicity Experiments with *D. magna*

Aerated tap water was used to prepare a serial concentration of BP or DEET solution. Ten *D. Magna* were put in a 100-mL glass beaker with 80 mL of solution. The experiments lasted 24 h. Each concentration test including cosolvent and blank control groups were designed with three parallel samples. The numbers of the dead individuals were recorded at the end of the test.

#### 2.3.3. Ninety-Six Hours Acute Toxicity Experiments with *B. rerio*

Aerated tap water was used to prepare a serial concentration of BP or DEET solution. Ten fish were put in a 5000-mL glass beaker with 4000 mL of solution. The experiments lasted 96 h. Each concentration test including cosolvent and blank control groups were designed with three parallel samples. The numbers of the dead individuals were recorded every 24 h.

#### 2.3.4. The Mixed Toxicity Experiment

The mixed toxicity experiments of BP and DEET were performed in the same method as the independent toxicity experiments. The ratios of BP and DEET used for *C. vulgaris*, *D. magna*, and *B. rerio* were different. All ratios of BP and DEET were randomly prepared, referred to the obtained EC_50_ or LC_50_ values of each independent chemical, in order to imitate different conditions in the water. 

Stock solutions were prepared for *C. vulgaris* (7.50 mg/L BPand 276.65 mg/L DEET), *D. magna* (50.10 mg/L BP and 347.20 mg/L DEET), and *B. rerio* (50.10 mg/L BP and 690.30 mg/L DEET). All stock solutions were set up as 100%; then, a series of solutions at different percentages were made for the test. When the EC_50_ or LC_50_ results are obtained as a percentage, the toxic units (TU) of BP and DEET can be calculated, respectively, by the following:

(1) $${\mathrm{TU}}_{i}=\frac{Ci}{EC50i}\;,$$

where C*_i_* was the concentration of *i* in the testing solution at the EC_50_ of the mixture.

TUi of BP + the TUi of DEET = the total TU of the mixture. If the total TU value at the endpoint of the mixture equaled 1, the toxicity was assumed to be additive; if the total TU value at the endpoint of the mixture was >1, the toxicity was assumed to have an antagonistic effect; if the total TU value at the endpoint of the mixture was <1, the toxicity was deemed to have a synergistic effect.

### 2.4. Statistical Analysis

OriginPro 9 software (OriginLab, Northampton, MA, USA) is used to draw graphs depicting the experimental results. IBM SPSS version 20 software (IBM, Armonk, NY, USA) was applied to calculate the 96-h half-maximal effective concentration (EC_50_) of *C. vulgaris*, the 24-h median lethal concentration (LC_50_) of *D. magna*, and 96-h LC_50_ of *B. rerio*.

### 2.5. Ecological Risk Assessment

AIST-MeRAM (National institute of Advanced Industrial Science and Technology-Multi-Purpose Ecological Risk Assessment and Management Tool, Japan, English ver. URL: http://en-meram.aist-riss.jp/) was employed to assess the ecological risk of the two chemicals. Two methods—assessment factor (AF) and species sensitivity distribution (SSD)—were used to deduce the predicted no-effect concentration (PNEC).

## 3. Results

There was no significant difference between the dimethyl sulfoxide control group at the highest concentration and the blank control group for *C. vulgaris* growth rate, *D. magna* lethal rate, and *B. rerio* lethal rate by analysis of variance. This indicated that dimethyl sulfoxide had no repression effect on *C. vulgaris*, *D. magna*, and *B. rerio* in this study. All the results were analyzed by comparing the testing to the blank control groups.

In the present study, the chemical concentrations were set up based on the results of the pre-test, so the concentration ranges are narrow and have good linear relation to the inhibition or mortality rates.

### 3.1. Acute Toxicity of BP

#### 3.1.1. *C. vulgaris* Growth Inhibition Test of BP

The inhibition effect on *C. vulgaris* growth increased with the increasing in the concentrations. During the exposure time, although the algal growth rate was inhibited, the algal densities still showed an increasing trend. When the BP concentration was 10.00 mg/L, there was almost no growth between 48 and 72 h, but rapid growth from 72 to 96 h.

**Figure 1:**
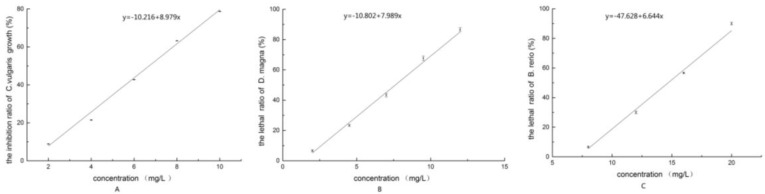
(**A**) The inhibition ratio of *C. vulgaris* growth of BP; (**B**) The lethal ratio of *D. magna* of BP; (**C**) The lethal ratio of *B. rerio* of BP.

The growth inhibition by BP concentration is shown in Figure 1A. The linear regression equation between BP concentration and its inhibition ratio was

**y** = −**10.216** + **8.979x**, (x: 2.00 mg/L~10.00 mg/L), (2)

where x is the BP concentration, and y is the inhibition ratio on *C. vulgaris* growth (%).

Using SPSS version 20 software, the 96-h EC_50_ was calculated as 6.86 mg/L (95% confidence interval: 6.40–7.34 mg/L). This value was classified as high-level toxicity on *C. vulgaris* [15].

#### 3.1.2. Acute Toxicity of BP to *D. magna*

When the concentrations range from 2.00 to 12.00 mg/L, the relationship between the mortality of *D. magna* and BP concentration was described by a linear equation:

y = −10.802 + 7.989x, (x: 2.00 mg/L~12.00 mg/L),
(3)

where x is the BP concentration, and y is the mortality of *D. magna* (%).

The 24-h LC_50_ of *D. magna* for BP was 7.63 mg/L (95% confidence interval: 7.13–8.14 mg/L), the value was also classified as high-level toxicity [15]. The acute toxicity of BP to *D. magna* is shown in Figure 1B.

#### 3.1.3. Acute Toxicity of BP to *B. rerio*

In a concentration range from 8.00 to 20.00 mg/L, the relationship between the mortality of *B. rerio* and BP concentration was described by a linear equation:

y = −47.628 + 6.644x, (x: 8.00 mg/L~20.00 mg/L),
(4)

where x is the BP concentration, and y is the mortality of *B. rerio* (%).

The acute toxicity of BP to *B. rerio* is shown in Figure 1C. The 96-h LC_50_ of *B. rerio* for BP was 14.73 mg/L (95% confidence interval: 14.11–15.39 mg/L), and this value was classified as medium-level toxicity [15].

### 3.2. Acute Toxicity of DEET

#### 3.2.1. *C. vulgaris* Growth Inhibition Test of DEET

**Figure 2:**
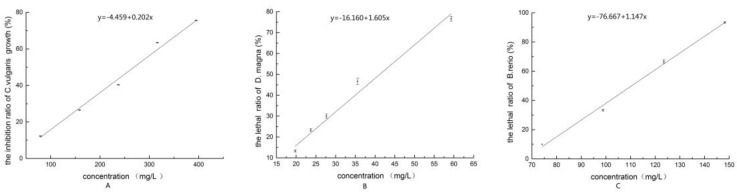
(**A**) The inhibition ratio of *C. vulgaris* growth of DEET; (**B**) The lethal ratio of *D. magna* of DEET; (**C**) The lethal ratio of *B. rerio* of DEET.

When the concentrations of DEET were between 79.04 and 395.20 mg/L, there was an obvious inhibition effect on *C. vulgaris*. With the increasing in the concentrations, the inhibition effect also increased. The *C. vulgaris* growth inhibition ratio by DEET concentration is shown in Figure 2A. The linear regression equation used was as follows:

y = −4.459 + 0.202x, (x: 79.04 mg/L~395.20 mg/L),
(5)

where x is the DEET concentration, and y is the inhibition ratio on *C. vulgaris* (%).

The 96-h EC_50_ was calculated as 270.72 mg/L (95% confidence interval: 250.40–292.50 mg/L) and showed low-level toxicity on *C. vulgaris* [15].

#### 3.2.2. Acute Toxicity of DEET to *D. magna*

The mortality of *D. magna* by DEET concentration was described by a linear equation:

y = −16.160 + 1.605x, (x: 19.76 mg/L~59.28 mg/L),
(6)

where x is the DEET concentration, and y is the mortality to *D. magna* (%). 

The 24-h LC_50_ for DEET was 40.74 mg/L (95% confidence interval: 37.86–44.25 mg/L), classified as medium-level toxicity [15]. The acute toxicity to *D. magna* of DEET is shown in Figure 2B.

#### 3.2.3. Acute Toxicity of DEET to *B. rerio*

The mortality of *B. rerio* by DEET concentration was described by a linear equation:

y = −76.667 + 1.147x, (x: 49.40 mg/L~148.20 mg/L),
(7)

where x is the DEET concentration, and y is the mortality to *B. rerio* (%). 

The acute toxicity to *B. rerio* of DEET is shown in Figure 2C. The 96-h LC_50_ of DEET was 109.67 mg/L (95% confidence interval: 106.14–141.15 mg/L) and was classified as low-level toxicity [15].

### 3.3. Mixed Acute Toxicity of BP and DEET

#### 3.3.1. Acute Toxicity of the Mixture to *C. vulgaris*

**Figure 3:**
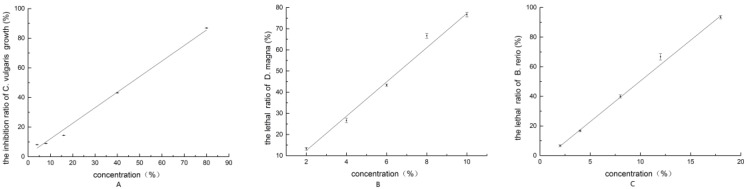
(**A**) The inhibition ratio of *C. vulgaris* growth by mixture; (**B**) The lethal ratio of *D. magna* by mixture; (**C**) The lethal ratio of *B. rerio* by mixture.

Based on the EC_50_ values of the independent toxicity experiments, the concentration of the mixture was calculated as a percentage of the total concentration (%). The relationship between the concentration of the mixture and the growth inhibition ratio of *C. vulgaris* is shown in Figure 3A. There was a good linear relationship between the concentration of the mixture and the growth inhibition ratio of *C. vulgaris*. The 96-h EC_50_ of the mixture of BP and DEET on *C. vulgaris* was 46.31% (95% confidence interval: 42.04%–51.21%). In this case, the concentration of BP in the mixture was 3.43 mg/L, and the concentration of DEET was 128.44 mg/L. By calculating the TU values of BP and DEET using Equation (1), respectively, the total TU value at this point of the mixture equaled 1. Therefore, the toxicity of BP plus DEET combination showed an additive effect [16].

#### 3.3.2. Acute Toxicity of the Mixture to *D. magna*

The mortality increased with the increasing in the concentration of mixture and showed a dose-response relationship. The acute toxicity of the mixture to *D. magna* is shown in Figure 3B.

The 24-h LC_50_ of the mixture of BP and DEET to *D. magna* was 6.61% (95% confidence interval: 6.11%–7.15%), the concentration of BP in the mixture was 3.31 mg/L, and the concentration of DEET was 22.95 mg/L. The total TU value at this point of the mixture equaled 1, so the BP plus DEET combination showed an additive effect [16].

#### 3.3.3. Acute Toxicity of the Mixture to *B. rerio*

The acute toxicity of the mixture to *B. rerio* is shown in Figure 3C. The mortality increased with the increasing in the concentrations and showed a dose-response relationship. 

The 96-h LC_50_ of the mixture of BP and DEET to *B. rerio* was 9.59% (95% confidence interval: 8.87%–10.36%), the concentration of BP in the mixture was 4.80 mg/L, and the concentration of DEET was 66.20 mg/L. The total TU value at this point of the mixture equaled 1, so the BP plus DEET combination showed an additive effect [16].

### 3.4. Assessment of the Acute Toxicity of BP and DEET

Two methods—the AF method and the SSD method—were used to deduce the PNEC values in the present study. The AF method uses the lowest toxicity data of fish, crustaceans including daphnids, and algae to characterize risk as a margin of exposure (MOE). This is a point risk estimate model for screening or initial risk assessment. The AF is divided by an uncertainty factor (UF) to deduce the PNEC value. The UF is different in different countries and organizations. In this study, the UF was selected as 100, which is recommended by the OECD.

The SSD method uses all of the available toxicity data to describe the differences in species sensitivity to chemicals as a statistical distribution. The HC5 (the concentration at which 5% of the species are affected) is derived from the dose-response curve of species sensitivity distribution. This is a quantitative and probabilistic risk estimate model.

In the present study, UF was suggested as 5 by the producer of the RAM-MeAIST, and the log-normal distribution (LND) estimation method was selected in the assessing results.

#### 3.4.1. Assessment of the Ecotoxicological Effect of BP

The values of 96-h EC_50_ of *C. vulgaris*, the 24-h LC_50_ of *D. magna*, and 96-h LC_50_ of *B. rerio* were applied to AIST-MeRAM. The deduced PNEC value by the AF method was 0.003 mg/L (using the uncertainty factor as 100), and was 0.480 mg/L by SSD (using the uncertainty factor as 5). The results including the former registered data are summarized in Table 1.

#### 3.4.2. Assessment of the Ecotoxicological Effect of DEET

The values of the 96-h EC_50_ of *C. vulgaris*, the 24-h LC_50_ of *D. magna*, and 96-h LC_50_ of *B. rerio* were applied to AIST-MeRAM. The deduced PNEC value by the AF method was 0.407 mg/L (using the uncertainty factor as 100) and was 8.494 mg/L by the SSD method (using the uncertainty factor as 5). The results, including the former studies, are given in Table 2.

## 4. Discussion

### 4.1. Assessment of the PNEC Values by AF and SSD

AF and SSD were employed in the present study to induce the PNEC. The values from SSD are 1–2 orders of magnitude higher than those from the AF method. To obtain reasonable results, at least 8 data (from different species or different tests) are needed for the SSD method, as shown in Table 1 and Table 2, there are 22 acute data from 8 species for BP, and 13 acute data from 9 species for DEET. Although the data of BP or DEET are more than 8, the data diversity and numbers were not enough for providing reasonable and trustworthy results, so the deduced value by SSD may underestimate the PNEC values. Therefore, the PNEC values deduced from the AF method are recommended and applied in the discussion for assessing the ecological risk of BP and DEET. We do not deny that, when the value from the AF method is applied as a reference to make criteria for protecting ecosystem, it may overestimate. However, from the point of protecting the ecosystem at the greatest extent, the PNEC value deduced by the AF method is more reasonable when there is no perfect evaluation system at present.

In the present study, the PNEC values of both chemicals were deduced by with and without the present data, respectively, the results show different characteristics. For BP, the PNEC values deduced by the AF method are the same values with or without the present data, while the results by the SSD method are a little different: 0.480 mg/L with present data and 0.443 mg/L without present data, respectively. For DEET, the PNEC values deduced by the AF method are 0.407 and 0.712 with and without the present data, respectively. The results by the SSD method are 8.494 mg/L with present data, but 10.00 mg/L without present data. Although it could not be concluded that these results increased or decreased the PNEC values, it is definitely that the present study enriched the database for the ecological assessment of the two chemicals and further improved the rationality of the assessment conclusion, for the principle of the assessment method is: the more data you provide, the more refined your assessment will be. 

### 4.2. The Acute Toxicity and Ecological Risk of BP

According to the acute toxicity results of BP, the 96-h EC_50_ for *C. vulgaris* was 6.86 mg/L, and the 24-h LC_50_ for *D. magna* was 7.63 mg/L. Both were judged as high-level toxicity. The 96-h LC_50_ for *B. rerio* was 14.73 mg/L, which was judged as medium-level toxicity. The sensitivity of *C. vulgaris* was higher than that of *D. magna* and *B. rerio*. To our knowledge, the present study is the first time to use *C. vulgaris* and *B. rerio* as organisms to test the acute toxicity of BP. The results provide new species and data for evaluation of the toxicity of BP.

Among all the acute toxicity data of BP registered in AIST-MeRAM, the 72-h EC_50_ of *Pseudokirchneriella subcapitata* (algae) was 3.50 mg/L, which was more sensitive than *C. vulgaris* in the present study. There were 4 data about the LC_50_ of *D. magna*; one was lower than 1.00 mg/L, the last three were all from 1.00 to 10.00 mg/L (7.60 mg/L, 10.00 mg/L, and 7.63 mg/L), which were similar to the present results. The 96-h LC_50_ of *Oryzias latipes* (fish) was 10.00 mg/L, which is a little lower than the results in the present study. There were 10 data about the LC_50_ of *Pimephales promelas* (fish), 9 of which were between 14.20 and 15.30 mg/L, which is very close to the 96-h LC_50_ value (14.73 mg/L) of *B. rerio* in the present study. These results indicated that the 96-h LC_50_ of BP tested by *B. rerio* was reasonable as compared with the results obtained by other species of fish. For the different trophic levels of biota exposed to BP, the sensitivities were different. Algae were more sensitive than fish. Algae are primary producers in the aquatic trophic web, the harmful effect on algae may affect the stability and function of the ecosystem, so the ecological risk of BP should be fully realized.

According to the assessment results by the AF method, the PNEC value of BP was 0.003 mg/L. It has been reported that the concentration detected in the Zhujiang River in China are 0.10–5.30 ng/L [17], and this value is much lower than the PNEC value. Similarly, the concentrations of the most commonly used UV filters, 4-MBC, BP-3, BP-4, and EHMC measured in coastal seawater samples from Spain ranged between 35 and 164 ng/L [18]. These facts indicated that BP could still be considered as a low environmental risk chemical at present. However, BPs have been detected in urine samples from individuals in the United States and China [3]. Concentrations of BP-3 (one of the derivatives of BP) have been measured at 9.97 mg/L in children and 15.7 mg/L in adults in United States; in China, the concentration of 0.62 mg/L in children and 0.98 mg/L in adults have been recorded. In Europe, concentrations of 4-MBC (4-methylbenzylidene camphor) and OC (octocrylene), two widely used UV filters, were determined in the muscle tissue of fish (brown trout, Salmo trutta fario) from seven small Swiss rivers, which all receiving inputs from wastewater treatment plants [19]. These indicated that the chemicals were transported into human and wild organism bodies through ecological processes and may have a potential health risk. BP has been proved as a type of endocrine disrupter, when it reaches an effective concentration, may cause damage to human health. However, the path of acquisition and how it affects the biota in the ecosystem still remain unknown. 

The results in the present study provided most recent ecotoxicological research for the BP. The acute toxic effects on tree typical organisms in the aquatic ecosystem were tested; further, the PNEC were deduced to evaluate the risk to environment. The results can provide references for environmental criteria. Because BPs are widely used in many personal care products in our everyday life, its potential hazard to aquatic organisms and to humans should be further studied.

### 4.3. The Acute Toxicity and Ecological Risk of DEET

In the acute toxicity results of DEET, the 96-h EC_50_ to *C. vulgaris* was 270.72 mg/L, the 24-h LC_50_ to *D. magna* was 40.74 mg/L, and the 96-h LC_50_ to *B. rerio* was 109.67 mg/L. The acute toxicity of *D. magna* was judged as medium-level toxicity, while the acute toxicity of *C. vulgaris* and *B. rerio* were judged as low-level toxicity. The sensitivity of *D. magna* was higher than that of *C. vulgaris* and *B. rerio*. To our knowledge, this is the first time to use *C. vulgaris* and *B. rerio* to test the acute toxicity of DEET. The results provided new data for the ecotoxicological assessment of DEET. 

Among all the data registered in the AIST-MeRAM about the acute toxicity of DEET, the 72-h EC_50_ to *Pseudokirchneriella subcapitata* (algae) was 100.00 mg/L, which was more sensitive than 96-h EC_50_ of *C. vulgaris*. There were three data of the acute toxicity of DEET to *D. magna*: 75.00 mg/L, 74.00 mg/L, and 40.74 mg/L. The present result (40.74 mg/L) is similar with these former studies, which indicated the rationality of the present result. There were two data of the LC_50_ of DEET to *Pimephales promelas* (fish)—both are 110.00 mg/L—and two data about the LC_50_ of DEET to *Gambusia affinis* (fish)—both are 235.00 mg/L. For *Oncorhynchus mykiss* (fish), the LC_50_ were 71.25 and 75.00 mg/L, respectively. There was only one LC_50_ of DEET to *Oryzias latipes* (fish) at 100.00 mg/L. The 96-h LC_50_ of *B. rerio* in this study is very close to all of the data reported on fish. According to the results for DEET described above, the sensitivity order is *Oncorhynchus mykiss* > *Daphnia magna* > *Oryzias latipes* = *Pseudokirchneriella subcapitata* > *Pimephales promelas* > *Gambusia affinis* > *Chlorella vulgaris*. Overall, the sensitivity of daphnids and fish was higher than that of algae. This can be explained that DEET is used as an insect repellent and there were corresponding target mechanisms in daphnids and fish.

The value of PNEC deduced by using the AF method is 0.407 mg/L. In the published papers, the concentrations of DEET detected in the aquatic environment ranged from 0.60 to 1.20 ug/L [20], which is lower than the PNEC value. Therefore, at present, DEET could be classified as chemicals having low toxicity and risk to the environment. To our knowledge, there are no reports about DEET in the human body; however, as DEET is used as repellents in some perfume, the human body cannot avoid being exposed to DEET. Furthermore, as a widely used ingredient in PCPs, it can be discharged to the aquatic environment so that they can reach humans directly or indirectly by processes of enrichment, accumulation, and amplification through the food web. The study of the acute toxicity of DEET can provide a scientific basis for criteria and thus for management of the exposure concentration and the discharge standard.

### 4.4. The Assessment of the Mixing Toxicity of BP and DEET

The mixture toxicity of BP and DEET on *C. vulgaris*, *D. magna*, and *B. rerio* in the present study all showed an additive effect. The results explained these two chemicals’ behavior in mixture conditions. Since BP and DEET have different structural compositions, their biological degradability pathways and degradation products may be different, so the interaction of the two chemicals afterwards in an actual environment may behave differently from the results in a laboratory. Therefore, the mixture of their degradation products may perform different interactions such as antagonism, additive effects, irrelevant effects, and synergy. 

The results of the present study provided the acute toxic responses of three tropical organisms in the aquatic ecosystem to BP, DEET, and their mixture. The acute toxic data enriched the tested species and acute testing results for the database, thereafter increasing the rationality of the induced PNEC. The PNEC induced from this study evaluated the ecological risk of both chemicals. These will provide a theoretical basis for establishing water quality criteria. 

In the future study, attention should be paid to the so-called lower risk, but may be potential disrupt chemicals. Further chronic toxic test and ecosystem level studies should also be carried to evaluate the physiological and developing effects on biota.

## 5. Conclusions

The acute toxic effects of BP and DEET on *C. vulgaris*, *D. magna*, and *B. rerio* were tested. The toxicity of BP to *C. vulgaris* and *D. magna* were judged as high-level toxicity, but that to *B. rerio* was judged as medium-level toxicity. For DEET, the acute toxicity of *C. vulgaris* and *B. rerio* were judged as low-level toxicity, and the acute toxicity of *D. magna* was judged as medium-level toxicity. The mixing toxicity of BP and DEET on *C. vulgaris*, *D. magna*, and *B. rerio* displayed an additive effect. The results explained the way of these two chemicals’ behavior in mixture conditions. The risk assessment to the environment by the AF method concluded that both chemicals are a low risk to the environment at present. The results in the present study indicated the diversity and complexity of the toxic effect of BP and DEET in PCPs.

Since both BP and DEET are main components in personal care products, they are therefore high exposure sources to humans. BP has been identified as endocrine disruptors [8], and their potential high risk to ecosystems and human health over a long time period should be made widely known. The assessment of the acute toxicity of BP and DEET in the present study clarified the different toxic levels and different sensitivities from different tropical organisms in the aquatic ecosystem, and the results indicated the diversity and complexity of the toxic effect of chemicals in PCPs. Although the BP and DEET are evaluated as low-risk chemicals in the aquatic ecosystem, the identified characteristic as an endocrine disruptor [8] implied the high potential risk of the PCPs over a long time period.

## Figures and Tables

**Table 1:** Acute toxicity result of benzophenone (including the former data registered in AIST-MeRAM).

Endpoint	Concentration (mg/L)	Exposure Duration (Days)	Species	Trophic Level	Source	Reference Number
EC_50_	3.50	3	*Unicellular green algae (Pseudokirchneriella subcapitata)*	Algae	Ministry of the Environment, Japan	323
EC_50_	6.86	4	*Chlorella vulgaris*	Algae	This study	
LC_50_	7.60	1	*Ceriodaphnia dubia*	Daphnids	ECOTOX	20160
EC_50_	0.28	1	*Daphnia magna*	Daphnids	ECOTOX	146689
EC_50_	10.00	2	*Daphnia magna*	Daphnids	Ministry of the Environment, Japan	323
LC_50_	7.63	1	*Daphnia magna*	Daphnids	This study	
LC_50_	15.30	4	*Fathead minnow (Pimephales promelas)*	Fish	EPA FHM	362
EC_50_	15.30	4	*Fathead Minnow (Pimephales promelas)*	Fish	EAT	5511
LC_50_	10.00	4	*Oryzias latipes*	Fish	Ministry of the Environment, Japan	323
EC_50_	13.70	1	*Pimephales promelas*	Fish	ECOTOX	23834
EC_50_	14.50	2	*Pimephales promelas*	Fish	ECOTOX	23835
EC_50_	14.90	1	*Pimephales promelas*	Fish	ECOTOX	23806
EC_50_	15.20	2	*Pimephales promelas*	Fish	ECOTOX	23807
LC_50_	14.20	4	*Pimephales promelas*	Fish	ECOTOX	116411
LC_50_	14.20	4	*Pimephales promelas*	Fish	ECOTOX	102620
LC_50_	14.50	2	*Pimephales promelas*	Fish	ECOTOX	23809
LC_50_	14.80	1	*Pimephales promelas*	Fish	ECOTOX	23808
LC_50_	15.20	1	*Pimephales promelas*	Fish	ECOTOX	51866
LC_50_	15.20	2	*Pimephales promelas*	Fish	ECOTOX	23805
LC_50_	15.30	4	*Pimephales promelas*	Fish	ECOTOX	102597
LC_50_	14.73	4	*Brachydanio rerio*	Fish	This study	

**Table 2:** Acute toxicity result of *N*,*N*-Diethyl-3-methylbenzamide (including the former data registered in AIST-MeRAM).

Endpoint	Concentration (mg/L)	Exposure Duration (Days)	Species	Trophic Level	Source	Reference Number
EC_50_	3.50	3	*Unicellular green algae (Pseudokirchneriella subcapitata)*	Algae	Ministry of the Environment, Japan	323
EC_50_	6.86	4	*Chlorella vulgaris*	Algae	This study	
LC_50_	7.60	1	*Ceriodaphnia dubia*	Daphnids	ECOTOX	20160
EC_50_	0.28	1	*Daphnia magna*	Daphnids	ECOTOX	146689
EC_50_	10.00	2	*Daphnia magna*	Daphnids	Ministry of the Environment, Japan	323
LC_50_	7.63	1	*Daphnia magna*	Daphnids	This study	
LC_50_	15.30	4	*Fathead minnow (Pimephales promelas)*	Fish	EPA FHM	362
EC_50_	15.30	4	*Fathead Minnow (Pimephales promelas)*	Fish	EAT	5511
LC_50_	10.00	4	*Oryzias latipes*	Fish	Ministry of the Environment, Japan	323
EC_50_	13.70	1	*Pimephales promelas*	Fish	ECOTOX	23834
EC_50_	14.50	2	*Pimephales promelas*	Fish	ECOTOX	23835
EC_50_	14.90	1	*Pimephales promelas*	Fish	ECOTOX	23806
EC_50_	15.20	2	*Pimephales promelas*	Fish	ECOTOX	23807
LC_50_	14.20	4	*Pimephales promelas*	Fish	ECOTOX	116411
LC_50_	14.20	4	*Pimephales promelas*	Fish	ECOTOX	102620
LC_50_	14.50	2	*Pimephales promelas*	Fish	ECOTOX	23809
LC_50_	14.80	1	*Pimephales promelas*	Fish	ECOTOX	23808
LC_50_	15.20	1	*Pimephales promelas*	Fish	ECOTOX	51866
LC_50_	15.20	2	*Pimephales promelas*	Fish	ECOTOX	23805
LC_50_	15.30	4	*Pimephales promelas*	Fish	ECOTOX	102597
LC_50_	14.73	4	*Brachydanio rerio*	Fish	This study	

## Abbreviations

The following abbreviations are used in this manuscript:

BP	benzophenone
DEET	*N*,*N*-Diethyl-3-methylbenzamide
PCPs	personal care products
PNEC	predicted no-effect concentration
